# The Distinctive Role of Gluconic Acid in Retarding Percutaneous Drug Permeation: Formulation of Lidocaine-Loaded Chitosan Nanoparticles

**DOI:** 10.3390/pharmaceutics16060831

**Published:** 2024-06-19

**Authors:** Amnon C. Sintov

**Affiliations:** 1 Department of Biomedical Engineering, Faculty of Engineering Sciences, Ben Gurion University of the Negev, Be’er Sheva 84105, Israel; asintov@bgu.ac.il; Tel.: +972-8-647-2709; 2 Laboratory for Biopharmaceutics, E.D. Bergmann Campus, Ben Gurion University of the Negev, Be’er Sheva 84105, Israel

**Keywords:** topical drug delivery, percutaneous permeation retardant, chitosan nanoparticles, gluconic acid, lidocaine

## Abstract

The objective of the present investigation was to evidence the skin retardation phenomenon of lidocaine by gluconic acid as an inactive ingredient involved in citrate-crosslinking chitosan nanoparticles. Lidocaine hydrochloride was loaded in nanoparticles based on chitosan, fabricated by using a water-in-oil microemulsion as a template and citric acid as an ionic cross-linker. Gluconic acid (pentahydroxy hexanoic acid) was added during the fabrication and compared with caproic acid, a non-hydroxy hexanoic acid. The chitosan nanoparticulate systems were characterized for mean particle size, particle size distribution, and zeta potential. The pentahydroxy hexanoic acid decreased the zeta potential to a significantly lower value than those obtained from both plain citrate and citrate–hexanoic acid formulations. The relatively lower value implies that gluconate ions are partly attached to the nanoparticle’s surface and mask its positively charged groups. It was also noted that the in vitro percutaneous permeation flux of lidocaine significantly decreased when gluconate-containing chitosan nanoparticles were applied, i.e., 6.1 ± 1.5 μg‧cm^−2^‧h^−1^ without gluconic acid to 3.4 ± 2.3 μg‧cm^−2^‧h^−1^ with gluconic acid. According to this result, it is suggested that gluconate ions played a role in retarding drug permeation through the skin, probably by calcium chelation in the *stratum granulosum*, which in turn stimulated lamellar body secretion, lipid synthesis, and intracellular release of Ca^2+^ from the endoplasmic reticulum.

## 1. Introduction

**Figure 1:**
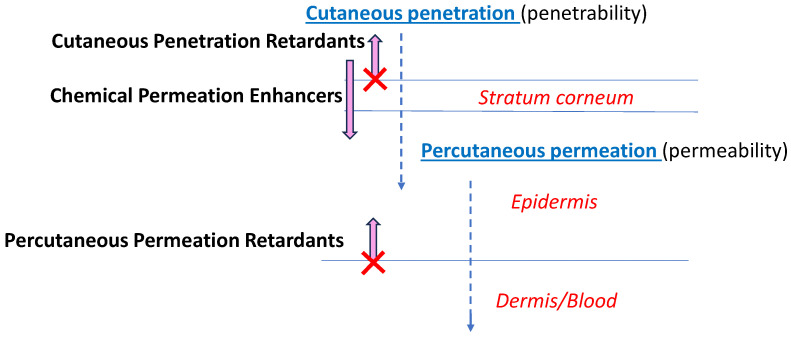
Penetration and permeation pathways of drugs into and through the skin, and the roles of CPEs (*chemical permeation enhancers*), CPRs (*cutaneous penetration retardants*, and PPRs (*percutaneous permeation retardants*) as modifiers of cutaneous and percutaneous drug delivery.

Nanoparticles (NPs) and other nanoscale carriers, such as microemulsions and nano-micelles, can facilitate drug delivery to the structural features of the skin, thus representing an alternative to the traditional dermal formulations [1]. The effective penetrability of nanoparticles across the upper skin barrier, the *stratum corneum*, is via one of three pathways: intracellularly through the corneocytes, intercellularly between corneocytes, or through hair follicles [1,2,3]. The *stratum corneum* is considered the main skin barrier, thus the enhanced penetration of the drug through this layer by nanosized formulations increases transdermal permeation and results in higher systemic delivery. However, transdermal permeation is not always guaranteed, as described in a previous paper [4] and illustrated in Figure 1. While *chemical permeation enhancers* (CPEs) and nanocarriers facilitate *stratum corneum* penetrability, potentially enabling drugs to be delivered through the skin, *cutaneous penetration retardants* (CPRs) and *percutaneous permeation retardants* (PPRs) reduce the fluxes of transdermal drug transport. Unlike CPRs that act by limiting drugs from passing through the *stratum corneum* (e.g., for products containing sunscreens and insect repellants), PPRs are chemical agents or formulations that increase the retention of drugs inside the skin compartment for the purpose of intradermal treatment.

Lidocaine HCl is a Skin-BCS (skin-related biopharmaceutics classification system) Class II drug [4], which is hydrophilic in nature with a low penetrability through the *stratum corneum*. However, the low penetrability of lidocaine can be overturned by using nanoparticles [4,5], as well as CPEs [6,7], or microemulsions [8,9,10]. In addition, a eutectic mixture of lidocaine and prilocaine (EMLA cream, Astra, Sweden) has also been commercially used as another solution for the low penetrability of lidocaine into the skin. In the previous publication [4], it has been shown that calcium-gluconate-based carbomer nanoparticles can retard lidocaine HCl permeation through the skin, while maintaining its penetrability into the skin and keeping a relatively high retention of the drug inside the skin. Thus, it has been clearly shown that the calcium gluconate salt, probably its gluconate ion, plays a role as a PPR when formulating it with carbomer nanoparticles. It has therefore been hypothesized that nanoparticles decorated with gluconate anions on their surface change the constant calcium gradient in the viable epidermis by Ca^2+^ depletion in the *stratum granulosum* [4]. This change in epidermal calcium gradient stimulates lamellar body secretion and lipid synthesis [11,12,13], as well as triggering endoplasmic reticulum Ca^2+^ release and desmosomal remodeling that, in turn, increase cell-to-cell adhesion [14,15]. In the present paper, more evidence is provided for the role played by gluconate ions in retarding the percutaneous permeation of lidocaine HCl in nanoparticles. Instead of using the anionic carbomer nanoparticles [4,16], cationic chitosan nanoparticles were manufactured by a W/O microemulsion crosslinking process. During this process, chitosan (CHS) was electrostatically crosslinked by sodium citrate and further reacted with gluconic acid δ-lactone (gluconolactone), which is instantly hydrolyzed in water to gluconic acid. The in-vitro percutaneous permeation and the skin retention of lidocaine applied in citrate-crosslinked chitosan/gluconate nanoparticles were examined in comparison to nanoparticles prepared without gluconic acid δ-lactone. Whereas lidocaine accumulation/retention in the skin did not change and remained relatively high, the percutaneous permeation flux of the drug significantly decreased when gluconate-containing chitosan nanoparticles were applied. As gluconic acid is a pentahydroxy-hexanoic acid, a comparison was also made with a non-hydroxy-hexanoic acid (caproic acid) in the citrate–chitosan NPs, showing a difference in the skin permeation rates of lidocaine after application of these lidocaine-loaded nanoparticles.

## 2. Materials and Methods

### 2.1. Materials

Chitosan was obtained from Sigma-Aldrich (Saint Louis, MO, USA), specified as follows: medium-molecular-weight polymer of 190,000–310,000 Da, 75% deacetylation level, and viscosity (1% in 1% acetic acid) of 200–800 cps. Isopropyl palmitate and propylene carbonate were purchased from Aldrich (Sigma-Aldrich Inc., Rehovot, Israel). Glyceryl oleate was obtained from Uniqema, Bromborough Pool, The Wirral, UK. Labrasol^®^ was obtained from Gattefossé, Saint-Priest, France. D-(+)-gluconic acid δ-lactone, caproic acid, lidocaine HCl, and sodium citrate were obtained from Sigma (Sigma-Aldrich Inc., Rehovot, Israel). Jaguar C 162^®^, a cationic derivative of hydroxypropyl guar (INCI name: hydroxypropyl guar hydroxypropyltrimonium chloride), was obtained from Solvay, France. Its specifications were as follows: substitution level ranged between 0.10 and 0.14 (cationic), viscosity at 25 °C was 300–100 mPa‧s, and it was a high-molecular-weight polymer (>400,000 Da) with a specific gravity of 700–850 kg/m^3^. High-performance liquid chromatography (HPLC)-grade solvents were obtained from J.T. Baker (Mallinckrodt Baker, Inc., Phillipsburg, NJ, USA).

### 2.2. Preparation of Nanoparticles

The polysaccharidic nanoparticles were manufactured using a microemulsion as a precursor. The microemulsion was prepared by mixing Labrasol^®^, glyceryl oleate (surfactants), and isopropyl palmitate (oil) with propylene carbonate (co-surfactant). The co-surfactant to surfactants (CoS/S) weight ratio was 1:5, and the surfactants’ ratio (i.e., glyceryl oleate/Labrasol ratio) was 1:3. Chitosan was first dissolved (10 mg/mL) in water containing citric acid at a concentration of 0.11 mmol/mL. Separate formulations were also prepared, in which gluconic acid δ-lactone or caproic acid (hexanoic acid) were added to the chitosan/citrate solution. Upon addition of gluconic acid δ-lactone, it instantly hydrolyzed to gluconic acid. The aqueous polymeric solution was solubilized in the oily phase, forming a microemulsion (3:7 W/O ratio). The obtained nano-sized dispersion contained 0.6 mg/g polymer and 0.0067 mmol/g citric acid (0.02 mmol/g COO^−^), and in separate formulations, an additional 0.02 mmol/g gluconic acid δ-lactone or an additional caproic acid (hexanoic acid) were incorporated in the spontaneously formed monophasic liquid. The system was then loaded with 10 mg/g lidocaine HCl and mixed until a clear liquid was obtained. A small quantity of 15 M sodium hydroxide solution was then added to adjust the pH to 5. Just for a comparison between the chitosan nanoparticles and a different cationic polymer-based nanoparticulate system in their topical lidocaine delivery, hydroxypropyl guar hydroxypropyltrimonium chloride (Jaguar C-162^®^) was selected. Jaguar C-162^®^ was first dissolved (2 mg/mL) in water containing citric acid at a concentration of 0.022 mmol/mL. A separate formulation was also prepared, in which gluconic acid δ-lactone was added into this cationic guar solution. The aqueous polymeric solution was solubilized in the oily phase, forming a microemulsion (3:7 ratio). The obtained nano-sized dispersion contained 0.6 mg/g polymer and 0.0067 mmol/g citric acid (0.02 mmol/g COO^−^). In the separate formulation, 0.02 mmol/g gluconic acid δ-lactone were incorporated in the spontaneously formed monophasic liquid. The system was then loaded with 10 mg/g lidocaine HCl and mixed for 5 min at 700 rpm until a clear liquid was obtained.

### 2.3. Nanoparticle Tracking Analysis (NTA)

Measurements were performed using a NanoSight NS300 instrument (Malvern Instruments Ltd., Malvern, UK), equipped with a 642 nm red laser module, 450 nm long-pass filter, and a camera operating at 25 frames per second, capturing a video file of the particles moving under Brownian motion. The software for capturing and analyzing the data (NTA 2.3 and 3.4) calculated the hydrodynamic diameters of the particles by using the Stokes–Einstein equation. Apart from measuring diameter, this instrument also measures the concentration of the particles.

### 2.4. Zeta (ζ) Potential (ZP)

ZP measurements were carried out using a Malvern Zetasizer (Nano ZS) instrument. The operation voltage was set to 40 V and a Malvern “folded capillary Cell” was used. Measurements (repeated three times) and analysis were carried out using Zetasizer 7.02 software, together with the Smoluchowski approximation for calculation of the ZP from the mobility measurements.

### 2.5. Skin Permeation Testing

#### 2.5.1. The Ex-Vivo Study

The penetration of lidocaine into the skin was determined in vitro using a Franz diffusion cell system (Permegear, Inc., Bethlehem, PA, USA). The diffusion area was 1.77 cm^2^ (15 mm diameter orifice), and the receptor compartment volumes were from 12 mL. The solutions in the water-jacketed cells were thermostated at 37 °C and stirred by externally driven, Teflon-coated magnetic bars. Each set of experiments was performed with at least four diffusion cells (n ≥ 4), each containing fresh abdominal rat skin. All animal procedures were performed in accordance with protocols reviewed and approved by the Institutional and Use Committee, Ben Gurion University of the Negev, which complies with the Israeli Law of Human Care and Use of Laboratory Animals, authorization number IL-30-06-2020 (C). Sprague–Dawley rats (males, 200–300 g) were euthanized by aspiration of CO_2_. The abdominal hair was carefully clipped, and sections of full-thickness skin were excised from the fresh carcasses of animals and used immediately. The skin was placed on the receiver chambers with the *stratum corneum* facing upwards, and the donor chambers were then clamped in place. The receiver chamber, defined as the side facing the dermis, was filled with phosphate buffer (pH 7.4) solution. After 15 min of skin washing at 37 °C, the buffer was removed from the cells and the receiver chambers were refilled with fresh phosphate buffer (pH 7.4)–ethanol (7:3) solution. The solubility of lidocaine (as a base) at pH = 7.4 is more than 35.2 μg/mL according to the Burnham Center for Chemical Genomics (https://pubchem.ncbi.nlm.nih.gov/bioassay/1996#section=Data-Table). Although a dilution process was made recurrently in the receiver chambers during every sampling time (see below), 30% ethyl alcohol was added to the receiver medium to keep a perfect sink condition. Aqueous dispersions (0.2 mL) of lidocaine-containing NPs, each conceived to contain 2 mg of entrapped lidocaine, were applied on the skin at time = 0. Samples (2 mL) were withdrawn from the receiver solution at one-hour intervals, and the receiver cell was replenished up to its marked volume with fresh buffer–ethanol solution each time. The receiver samples were taken into 1.5 mL vials and kept at −20 °C until analyzed by HPLC.

#### 2.5.2. Skin Integrity Testing

All skin sections were measured for transepidermal water loss (TEWL) and only those pieces with TEWL levels less than 10 g/m^2^/h were used. TEWL testing was performed on skin pieces using a Dermalab Cortex Technology instrument, (Hadsund, Denmark).

#### 2.5.3. Skin Extraction

After a 6 h experimental period, each exposed skin tissue was washed with plenty of water, wiped carefully and tape stripped (×15) to remove lidocaine adsorbed in the *stratum corneum*. The washed skin tissue was cut into small pieces, inserted into 2 mL vials, and extracted by 1 mL ethanol. The extraction was performed by incubation in a shaker (750 rpm) for 60 min. After centrifugation, the extracts were taken into 1.5 mL vials and kept at −20 °C until analyzed by HPLC within two days.

### 2.6. HPLC Analysis of Samples

Aliquots of 20 mL from each sample were injected into a HPLC system, equipped with a prepacked column (Betasil™ C18, 5 µm, 250 × 4.6 mm, Thermo Scientific, Morecambe, UK). The HPLC system (1260 Infinity II, Agilent Technologies Inc., Santa Clara, CA, USA) consisted of an auto-injector and a diode array detector. The quantification of lidocaine was carried out at 210 nm. The samples were chromatographed using an isocratic mobile phase consisting of 0.02 M phosphate buffer solution pH 6.0/acetonitrile (35:65) at a flow rate of 1 mL/min. A linear calibration curve (peak areas versus drug concentrations over the range of 0.5–50 μg/mL) was constructed by running standard drug solutions for each series of chromatographed samples. The retention time of the lidocaine peak was at 6.5 min and its specificity were ascertained by using an in vitro skin permeation test of an unloaded nanoparticle dispersion as a blank/placebo control. The limit of detection (LOD) was 0.1 μg/mL, while the limit of quantification (LOQ) was 0.5 μg/mL.

### 2.7. Calculation of Lidocaine Permeation through the Skin

As a result of the sampling of large volumes from the receiver solution (and their replacement with equal volumes of buffer), the receiver solution was constantly being diluted. Taking this process into account, the cumulative drug that permeated out into the receiver ($Q_{out}\left(t_{n}\right)$) at the end of the nth sampling time (n≥0) was calculated according to the following equation:

(1) $$Q_{out}\left(t_{0}\right)=C_{out}\left(t_{0}\right)=0\;\;\;;\;\;\;\;t_{0}^{-}=t_{0}=0$$

(2) $$Q_{out}\left(t\right)=V_{r}\;C_{out}\left(t_{n}^{-}\right)+\sum_{i=0}^{n-1}Vs\;C_{out}\left(t_{n}^{-}\right)\;\;n\geq 1$$

(3) $$C_{out}\left(t_{n}\right)=\left[C_{out}\left(t_{n}^{-}\right)\cdot \left(V_{r}-V_{s}\right)\right]/V_{r}$$

where $C_{out}\left(t_{n}\right)$ is the drug concentration in the receiver at sampling time $t_{n},$ expressed by a running number $\left(t=1,\;2,\;3\ldots t_{n}\right)$. $V_{r}$ and $V_{s}$ are the constant volumes of the receiver and the sample solutions, respectively. Data were expressed as the cumulative drug permeation per unit of membrane surface area, $Q_{out}\left(t_{n}\right)$/*S* (*S* = 1.77 cm^2^). The steady-state fluxes (*Jss*) were calculated by linear regression interpolation of the experimental data at a steady state:

(4) $$Jss=∆Q_{out}\left(t_{n}\right)/(∆t_{n}\cdot S)$$

### 2.8. Statistical Analysis

The statistical differences between the percutaneous permeation data obtained from the various formulations were analyzed, employing the two-way unweighted means analysis of variance (ANOVA) test. The differences among groups were considered significant when *p* values < 0.05.

## 3. Results

### 3.1. Nanoparticle Characterization

**Figure 2:**
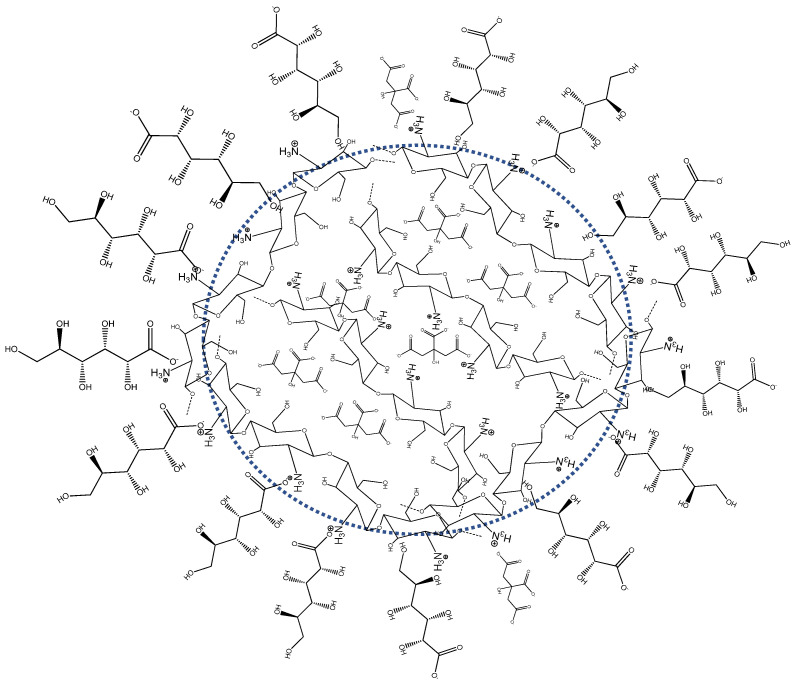
Schematic representation of citrate-chitosan nanoparticles. Due to their pentahydroxy groups, gluconate molecules partially bind onto the polysaccharidic particle surface via hydrogen bonds and not through the ionic carboxylate groups only.

In a similar way to the carbomer nanoparticles’ mode of preparation [4], chitosan-based nanoparticles (CHS-NPs) were prepared using a W/O microemulsion as a template. Crosslinking of chitosan in the inner aqueous phase of the microemulsion was performed by citrate ions, which modifies the properties of chitosan nanoparticles (e.g., increased molecular weight, increased polymer solidity, decreased water solubility). Table 1 summarizes the mean particle size, particle size distribution, and ζ potential of three citrate-crosslinked chitosan nanoparticulate systems, with and without monocarboxylic acids—hexanoic acid (caproic acid), and pentahydroxy-hexanoic acid (gluconic acid). As is shown, the distribution and the mean particle size of the various chitosan nanoparticles were similar, ranging between 96–107 nm (D10) and 235–269 nm (D90), while the mean size was between 155 nm and 177 nm. Nanoparticle concentration was found to be between 2.26 × 10^13^ particles/mL and 3.12 × 10^13^ particles/mL. Nevertheless, the zeta (ζ) potential values were found to be different between the formulations. Plain citrate-crosslinked NPs had a zeta potential of 29.5 mV, whereas the addition of hexanoic acid increased the potential to 35.4 mV. This may imply that the fatty-acid-derived monocarboxylate ions competed with the tricarboxylic citrate ions, resulting in more free, charged, quaternary ammonium cations distributed on the particle surface. In contrast to the hexanoic acid, the pentahydroxy-hexanoic acid decreased the zeta potential to 23.0 mV, a significantly lower value than those obtained from both plain citrate and citrate–hexanoic acid formulations. The relatively lower value implies that gluconate ions are partly attached to the nanoparticle’s surface and mask its positively charged groups. This interesting finding is also in agreement with the previous publication [4], which showed that the zeta potential of negatively charged carbomer nanoparticles changed from −36 mV to −23.3 mV due to partial masking of the abundant charged groups on the surface by gluconate ions (calcium gluconate). It was hypothesized that gluconate ions partially coated the nanoparticle surface through hydrogen bonding, and partially bound through calcium bridges. This hypothesis can also be relevant to the current study, and is even supported by using a different system of positively charged chitosan nanoparticles containing gluconate ions (Figure 2).

As illustrated in Figure 2, gluconate ions can be fused into the CHS-NPs in two ways: (a) formation of ionic bonds, i.e., binding via their carboxylate anions to the quaternary ammonium cations on chitosan chains, thus competing with citrate ions on the binding sites; and (b) formation of hydrogen bonds, i.e., binding via dipole–dipole attraction between their multiple hydroxyl groups to the hydroxyl groups on the polysaccharidic chains of chitosan.

### 3.2. Skin Permeation of Lidocaine

**Figure 3:**
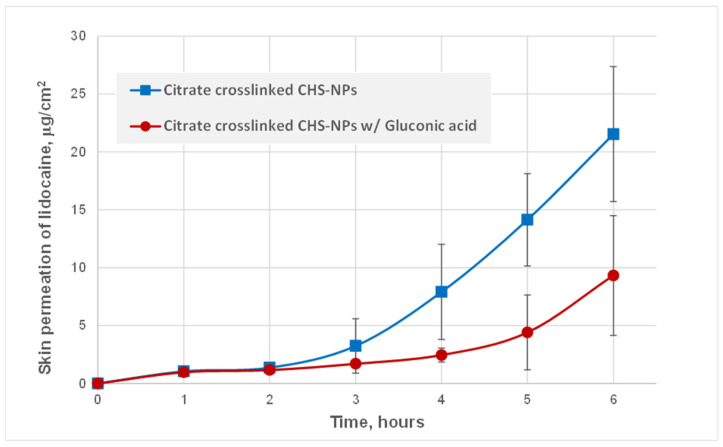
Percutaneous permeation kinetics of lidocaine applied in citrate-crosslinked CHS-NPs and in citrate-crosslinked CHS-NPs/gluconic acid combination.

Figure 3 presents the permeation profiles of lidocaine from citrate–chitosan nanoparticles across freshly excised rat skin. A significant difference was obviously noted between citrate-crosslinked CHS-NPs with and without the incorporation of gluconic acid (ANOVA, *p* < 0.05), suggesting that gluconate ions played a role in retarding drug permeation through the skin. After a lag time that lasted approximately 3 h, the mean permeation flux of lidocaine in citrate-crosslinked CHS-NPs was 6.1 ± 1.5 μg‧cm^−2^‧h^−1^, while the flux of the drug applied in the citrate-crosslinked CHS-NPs/gluconic acid combination was relatively 2-fold lower, 3.4 ± 2.3 μg‧cm^−2^‧h^−1^. After 6 h of the diffusion process, 21.5 ± 5.8 μg lidocaine per cm^2^ of skin surface area permeated and accumulated in the receiver compartment after its application with citrate-crosslinked CHS-NPs, whereas only 9.3 ± 5.2 μg/cm^2^ of lidocaine was transported through the skin after its application with the citrate-crosslinked CHS-NPs/gluconic acid combination. Lidocaine skin permeation after its application in the citrate-crosslinked CHS-NPs/caproic acid combination was even higher than that measured after application of the plain citrate-crosslinked CHS-NPs (ANOVA, *p* < 0.05). The mean permeation flux of lidocaine in the citrate-crosslinked CHS-NPs/caproic acid combination was 8.3 ± 1.6 μg‧cm^−2^‧h^−1^ after a lag time of 2 h, and the cumulative quantity permeated through the skin after 6 h was 36.6 ± 8.6 μg/cm^2^. This finding may suggest that caproic acid (a) decreases the crosslinking level by competing with citrate ions over the cationic groups on the CHS chain, thus increasing lidocaine release from the nanoparticles, and/or (b) enhances drug permeation due to its amphiphilic property as a fatty acid, being capable of intercalating with skin lipid bilayers [17].

The significantly higher permeation rates of plain citrate-crosslinked CHS-NPs and the citrate-crosslinked CHS-NPs/hexanoic acid combination compared to the citrate-crosslinked CHS-NPs/gluconic acid combination, emphasizes the distinctive gluconate-derived structure of the latter nanoparticles, which leads to drug retardation in the skin tissues. Lidocaine retention in the epidermis–dermis layers were analyzed 6 h after application, demonstrating that equal cumulative quantities of lidocaine exist in the skin tissues of both plain citrate-crosslinked CHS-NPs and the citrate-crosslinked CHS-NPs/gluconic acid combination, at 21.3 ± 7.7 μg‧cm^−2^ and 20.8 ± 8.0 μg‧cm^−2^, respectively. This may indicate that the drug retardation caused by the citrate-crosslinked CHS-NPs/gluconic acid combination is expressed as a steeper gradient of lidocaine from the epidermis to the dermis, rather than the more moderate gradient created by the plain citrate-crosslinked CHS-NPs.

**Figure 4:**
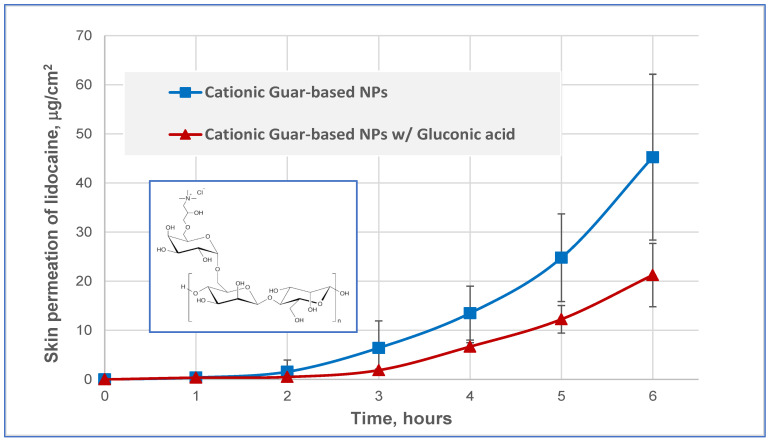
Percutaneous permeation kinetics of lidocaine applied in cationic guar NPs and in cationic guar NPs/gluconic acid combination.

Although the use of cationic chitosan-based NPs has clearly shown that the addition of gluconate retarded the skin permeation of lidocaine and played a role of a PPR, it was conceived that a different cationic polymer might be required for validating and confirming this phenomenon. The cationic polymer, hydroxypropyl guar hydroxypropyltrimonium chloride (Jaguar C-162^®^), is a quaternary ammonium derivative of guar gum used in shampoos and hair conditioning products. Lidocaine-containing nanoparticles were fabricated with this polymer with and without 0.02 mmol/g gluconic acid. Figure 4, which presents the cumulative percutaneous permeation of lidocaine after application of these nanoparticle suspensions, demonstrates drug retardation of the gluconate-containing system compared to the plain cationic guar nanoparticles. After a lag time that lasted approximately 2.5–3 h, the mean permeation flux of lidocaine in plain cationic guar NPs was 12.8 ± 5.9 μg‧cm^−2^‧h^−1^, while the flux of the drug applied in the cationic guar NPs/gluconic acid combination was relatively 2-fold lower, 6.4 ± 2.0 μg·cm^−2^·h^−1^. This 2-fold decrease in the permeation rate of lidocaine after application of the cationic guar NPs/gluconate combination is consistent with the 2-fold decrease obtained after using the CHS-NPs/gluconate combination.

### 3.3. Mechanistic Explanation of the Skin Drug Retardation by Gluconate-Containing NPs

**Figure 5:**
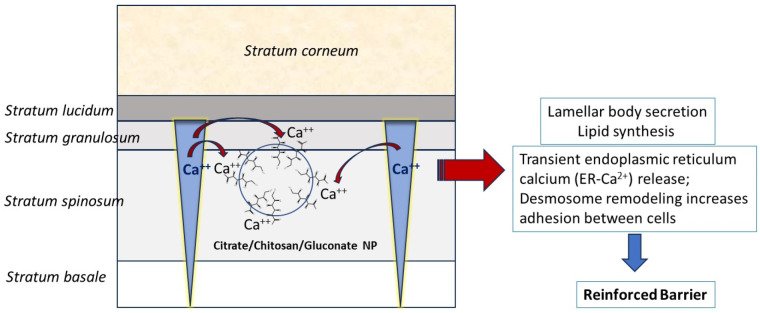
Proposed mechanism of reinforcing the skin barrier without its pre-impairment, by the citrate-crosslinked CHS-NPs/gluconate combination.

There is growing evidence that the viable epidermis also functions as a skin barrier, being a second line of defense against external factors, such as disruption or perturbation of the *stratum corneum*, and sonophoresis or iontophoresis. The skin barrier mechanism of the viable epidermis includes lamellar body secretion from the *stratum granulosum* [11,18,19], increased lipid synthesis [12,13], release of intracellular endoplasmic reticulum (ER) calcium [14], and remodulation of desmosomes that increases cell adhesion [15]. These activities are abruptly provoked when the extracellular Ca^2+^ gradient, which normally exists from the *stratum granulosum* (high levels) to the *stratum basalis* (low levels), is lost due to barrier disruption and calcium depletion [18,19]. It has been proposed following the previous research [4] and the present study that NPs decorated by the free carboxyl ion groups of gluconic acid, may change the constant calcium gradient by chelating Ca^2+^ ions. Figure 5 presents how citrate-crosslinked CHS-NPs with protruding gluconate groups can deplete Ca^2+^ from the *stratum granulosum*, thus changing the calcium gradient. According to the mechanism schematically illustrated in Figure 5, the skin barrier is fortified following the disrupted epidermal calcium gradient, which in turn, results in retardation of the percutaneous permeation of drugs.

## 4. Conclusions

The major goal of dermatological and cosmeceutical treatment is local delivery and targeting drugs and active agents to the skin tissues. The targeted delivery increases the in-situ activity of the active agent and limits adverse effects resulting from systemic exposure. Nanocarriers and CPEs enable drug penetration through the *stratum corneum*, which is the main skin barrier, but cannot prevent its transdermal permeation and transport to the blood circulation. To achieve the major goal of topical dermatological treatment, the secondary barrier of the skin, epidermal bioprocessing, is to be exploited. This secondary barrier includes stimulation of lamellar body secretion from granular cells and ER Ca^2+^ release, driven by calcium gradient disruption. It is therefore proposed, according to the present study, that a topical nanoformulation with protruding gluconic acid as a PPR will be used. Concomitant with a previous publication describing the anionic carbomer NPs/calcium gluconate combination [4], the current study has made it obvious that decoration of gluconate ions on polymeric NPs, such as the cationic chitosan-based NPs, resulted in retardation of the percutaneous permeation of lidocaine, probably by chelation of calcium ions in the skin and disrupting the epidermal calcium gradient. A full characterization of the citrate-crosslinked chitosan NPs that were applied in this study is beyond the scope of the present paper; however, it certainly remains to be carried out and published in separate research.

## Figures and Tables

**Table 1:** Schematic representation of citrate–chitosan nanoparticles. Due to their pentahydroxy groups, gluconate molecules partially bind onto the polysaccharidic particle surface via hydrogen bonds and not through the ionic carboxylate groups only.

	Citrate-Crosslinked CHS-NPs	Citrate-Crosslinked CHS-NPs w/Gluconic Acid	Citrate-Crosslinked CHS-NPs w/Caproic Acid
Mean particle size (nm)	159.5	177.0	155.2
PDI	0.22	0.20	0.18
D10 (nm)	96.5	107.4	98.0
D50 (nm)	138.1	155.9	135.0
D90 (nm)	239.2	269.0	235.8
NP concentration (NPs/mL) (±SD)	2.76 × 10^13^ (±1.49 × 10^12^)	2.26 × 10^13^ (±6.36 × 10^11^)	3.12 × 10^13^ (±2.98 × 10^12^)
ζ potential (±SD) (mV)	29.5 (±0.5)	23.0 (±1.00)	35.4 (±1.0)

## Data Availability

The data presented in this study are available in this article.
